# Facile Enhancement of Corrosion, Wear and UV Aging Resistance of Epoxy Composite Coatings via Graphene Oxide Incorporation

**DOI:** 10.3390/nano16191275

**Published:** 2026-10-08

**Authors:** Fujun Zhang, Boyi Qiu, Xusheng Du

**Affiliations:** 1 School of Civil Engineering, Lanzhou Jiaotong University, Lanzhou 730070, China; 13250048@stu.lzjtu.edu.cn; 2 Gansu Changda Highway Co., Ltd., Lanzhou 730000, China; 3 Institute of Advanced Wear & Corrosion Resistant and Functional Materials, Jinan University, Guangzhou 510632, China; qby123@stu2024.jnu.edu.cn; 4 National Joint Engineering Research Center of High Performance Metal Wear Resistant Materials Technology, Jinan University, Guangzhou 510632, China

**Keywords:** graphene oxide, epoxy composite coating, UV aging, corrosion resistance, wear, thermal cycling, water uptake

## Abstract

UV aging, corrosion and wear resistance of epoxy (EP) coatings are all critical for their long-term service in outdoor environments. In this work, graphene oxide (GO) was used as a multifunctional additive in epoxy coatings to improve these properties. Owing to its two-dimensional sheet-like nanostructure and abundant oxygen-containing functional groups, which enable a favorable interface with the epoxy matrix, incorporating GO into epoxy coatings endows the coating with multiple enhanced performances, including anti-corrosion performance, UV resistance, wear resistance, and adhesion to metal substrates. The resulting GO/EP composite coating exhibits higher adhesion strength (15.08 MPa) than that of neat epoxy coating (12.02 MPa), and delivers excellent corrosion resistance, with a corrosion current density of 4.69 × 10^−11^ A·cm^−2^ which is as low as 1/3 that of the neat epoxy coating after 25 days of immersion in NaCl solution. Notably, after multiple cycles of thermal cycling testing, the coating maintains an impedance ~40 times higher than that of neat epoxy coating. After UV aging testing, comparing to neat EP coating, the GO/EP coating experiences far less degradation in gloss, water resistance, corrosion resistance and bonding strength to steel substrates. In addition, tribological tests confirmed that GO/EP has improved wear resistance compared with neat epoxy coating and the wear rate of epoxy coating was reduced by ~30% with incorporation of only 0.1%wt GO. This study provides a promising strategy for developing durable protective coatings for practical applications.

## 1. Introduction

As one of the most widely used material categories, metals are applied across numerous fields, including civil engineering, marine engineering and aeronautical engineering. Corrosion of metal structures occurs commonly in certain environments, leading to structural failure and even severe safety risks. Therefore, the research and application of coatings with long-term, reliable protective performance is highly significant [1,2,3]. Thanks to their excellent adhesion, chemical resistance and mechanical properties, epoxy coatings have been widely adopted for metal protection [4,5,6]. However, conventional epoxy coatings mainly rely on physical barrier effects to block the penetration of corrosive substances, and their long-term protective performance still requires improvement.

Due to their excellent chemical stability, high electrical conductivity, environmental friendliness and large specific surface area, carbon nanomaterials have been widely used as functional fillers in polymer composite coatings. Common carbon nanomaterials include carbon nanotubes (CNTs), graphene and graphene foam. CNTs have been applied to improve the anti-corrosion properties of coatings [7,8], and epoxy composite coatings containing 2 wt% CNTs have demonstrated the optimal anticorrosion performance [8]. Graphene films have excellent impermeability to corrosive media and have been developed as high-performance coatings to protect various metals [9,10,11]. Meanwhile, as a functional filler, graphene can endow polymer composite coatings with better barrier performance at a much lower loading [12,13,14,15,16,17] compared with CNTs. Recent studies have shown that the corrosion current intensity (I_corr_) of zinc-rich epoxy coatings modified with 0.2 wt% 3D graphene is approximately 6 orders of magnitude lower than that of the counterpart modified with CNTs at the same loading [18].

Graphene oxide (GO) is an important derivative of graphene. It features a two-dimensional layered structure, high aspect ratio, and abundant oxygen-containing functional groups, making it a promising functional candidate for anti-corrosion applications [19,20,21,22,23,24,25,26,27,28]. When incorporated into polymer coatings, GO can form tortuous diffusion pathways, which enhances the resistance to the intrusion of water, oxygen and corrosive ions. Recently, it has been reported that after introducing 0.3 wt% GO into zinc-rich epoxy coatings, the I_corr_ of the coating was reduced by approximately 5 orders of magnitude [25].

Moreover, in the actual application scenarios of metal infrastructure, such as highway bridges and power transmission towers, thermal cycling, ultraviolet (UV) aging, and surface wear or scratching of the protective coating all require close attention to extend its service life. Under UV irradiation, macromolecules in the coating may undergo chain scission, which causes degradation of the coating’s surface protective performance, including reduced gloss, chalking, accelerated corrosion, and weakened mechanical properties. To develop UV-resistant coatings, special chemical additives such as cerium-based oxides have been incorporated into polymer composite coating systems [29,30,31,32], since these additives can eliminate UV-induced free radicals and slow down the photodegradation of the polymer matrix. Recent research has shown that after 24 h of UV aging and 12 days of immersion in NaCl solution, the epoxy coating modified with CeZrOx@BN nanosheet hybrid filler still maintains a low-frequency impedance eight times higher than that of the unmodified control sample [33].

As is well known, carbon nanomaterials including carbon nanotubes, graphene, and carbon dots exhibit good barrier performance against UV irradiation, and are also recognized as excellent radical scavengers due to their strong affinity for free radicals. However, for practical industrial applications, current understanding of their contribution to the comprehensive surface protection performance of epoxy coatings, including thermal cycling resistance, UV aging resistance and corrosion resistance, is still far from sufficient. Accordingly, in this study, GO was used as a multifunctional filler to prepare an epoxy composite coating for steel surface protection. This fully distinguishes the present study from our previous work [25,34], where GO was incorporated to improve the corrosion resistance of zinc-rich epoxy coatings. In the previous work, the cathodic protection provided by zinc microplates in the epoxy matrix worked in synergy with the barrier properties of GO. The effects of GO incorporation on the anticorrosion performance, thermal cycling resistance, UV resistance and wear resistance of the epoxy coating, as well as the coating’s adhesion to the substrate, were systematically evaluated.

## 2. Materials and Methods

### 2.1. Materials

E-44 epoxy resin was purchased from Xiya Chemical Technology (Shandong) Co., Ltd., (Linyi, China). Graphene oxide (GO) and Q235 carbon steel panels were procured from Suzhou Tanfeng Graphene Technology Co., Ltd., (Suzhou, China) and Fuquan Metal Co., Ltd., (Qingdao, China), respectively.

### 2.2. Preparation of GO/EP Modified Coating

A mixed solvent consisting of 0.15 g of n-butanol and 0.35 g of xylene was prepared as the epoxy diluent. 3 mg of GO was dispersed in a mixture of 0.15 g n-butanol and 0.35 g xylene, followed by ultrasonication for 30 min (160 W, KQ-800KDE digital ultrasonic cleaner, Kunshan Ultrasonic Instrument Co., Ltd., Kunshan, China). Subsequently, 2 g of epoxy resin was introduced and stirred for 4 h, followed by the addition of 1 g of polyamide 651 curing agent (curing agent-to-epoxy resin mass ratio of 1:2) with continuous stirring for 10 min to obtain the GO/EP coating formulation. Q235 steel panels were sequentially abraded with sandpapers, degreased with ethanol under ultrasonication, and cleaned. The coatings were uniformly applied using a film applicator and cured at 60 °C for 24 h. The dry film thickness of the coatings is 80 ± 5 μm. The GO/EP coating with different content of GO was prepared and the content of GO in the coating is defined as the mass fraction of GO relative to the total mass of the epoxy resin (E44) and the polyamide 651 curing agent. A neat EP coating without any filler was used as a blank reference.

### 2.3. Characterization

#### 2.3.1. Materials Characterization

The surface morphology of the samples were observed using field-emission scanning electron microscopy (SEM, ULTRA55, Carl Zeiss, Oberkochen, Germany). The crystal structure of the samples were characterized by X-ray diffraction (XRD, Ultima IV, Rigaku, Japan) with Cu Kα radiation (λ = 0.154 nm) at a working voltage of 40 kV and a current of 40 mA, with a scanning rate of 5°·min^−1^. Thermal stability of GO and its epoxy composites were evaluated by thermogravimetric analysis (TGA) under nitrogen atmosphere from room temperature to 600 °C at a heating rate of 10 °C·min^−1^.

#### 2.3.2. Thermal Cycling Resistance Test

The service stability and protective performance evolution of the coatings under alternating temperature conditions were evaluated using an alternating temperature test chamber. Each thermal cycling process consisted of 12 h of holding at −30 °C followed by 12 h of holding at 60 °C, with the heating and cooling procedures automatically controlled by the instrument’s built-in program. Neat EP-coated and GO/EP-coated steel samples were placed in the chamber at the same time to undergo cyclic testing and a total 10 cycles were conducted on the same specimen. After each cycle, the specimen was taken out, allowed to equilibrate to room temperature, then tested via electrochemical impedance spectroscopy (EIS) in 3.5 wt.% NaCl solution, to track how the impedance performance changed with the number of cycles.

#### 2.3.3. Corrosion Protection Performance Evaluation

The anticorrosion performance of all coatings was evaluated using a Gamry (1010e) electrochemical workstation with a three-electrode system, in which the coated Q235 steel panel (exposed area of 1 cm^2^) served as the working electrode, a saturated calomel electrode (SCE) as the reference electrode, and a platinum sheet as the counter electrode. The corrosive medium was 3.5 wt.% NaCl aqueous solution. The corrosion protection performance was assessed through both EIS and potentiodynamic polarization (also named as ‘Tafel’) measurements. EIS tests were conducted after the open circuit potential reached a stable value, over a frequency range of 100 kHz to 10 mHz with an AC amplitude of 5 mV. The raw EIS data were fitted using an equivalent circuit model with ZView 3.0 software. Potentiodynamic polarization scans were performed from −0.5 V to +0.5 V relative to the corrosion potential at a scan rate of 10 mV·s^−1^. For long-term immersion tests, EIS test of the EP or GO/EP coated samples immersed in 3.5 wt.% NaCl solution was performed with periodic duration up to 25 days.

#### 2.3.4. UV Aging Test

UV-accelerated aging tests were conducted performed according to GB/T 1865–2009 using a BGD 865-A benchtop xenon lamp aging test chamber using a filtered xenon-arc lamp (daylight filter) (Guangzhou Biaogeda Experimental Instrument Co., Ltd., Guangzhou, China). The sample-to-lamp distance was 220 mm. The irradiation intensity was set at 60 W·m^−2^, and the black-panel temperature was maintained at 60 ± 2 °C. The steel specimens coated with either EP or GO/EP were subjected to UV aging for 100 h and their EIS tests were performed following the aforementioned conditions.

#### 2.3.5. Adhesion Strength Measurement

The adhesion strength of the coatings to the metal substrate was measured using the pull-off method in accordance with GB/T 5210-2006 [35]. Aluminum cylindrical loading fixtures (20 mm in diameter) were vertically adhered to the coating surface using an epoxy resin adhesive. After curing at room temperature for 24 h, a universal testing machine (UTM5000, Shenzhen Sansi Zongheng Technology Co., Ltd., Shenzhen, China) was employed to apply a tensile load at a uniform rate of 0.5 mm·min^−1^ until the coating was detached from the substrate. The maximum tensile stress (MPa) at the point of failure was recorded as the adhesion strength.

#### 2.3.6. Wear Resistance and Tribological Test

The friction and wear performance of the coatings were evaluated according to ASTM G133-22 [36] using a tribometer (MFT5000, Rtec Instruments, Nanjing, China) under reciprocating sliding mode with a reciprocating distance of 10 mm. Gwyddion image analysis software (version 2.62) was used to analyze the cross-section of the wear scar and the wear volume of the sample. A GCr15 steel ball with a diameter of 5 mm was used as the counterpart. The specimens were cleaned with ethanol and dried prior to testing. The tests were conducted at a frequency of 2 Hz under an applied load of 5 N for a duration of 30 min. Each test was repeated at least three times under identical conditions to ensure reliability and reproducibility. The scratch depth and wear volume were measured using a surface profilometer, and the wear volume (Δ*V*) was determined accordingly. The wear rate (*W*) was calculated according to the following equation:

$$W=\frac{\Delta V}{\mathrm{FL}}$$

where *W* is the wear rate, m^3^/(N·m); Δ*V* is the wear volume, m^3^; *F* is the applied load (N); *L* is the total sliding distance, m.

## 3. Results and Discussion

### 3.1. Structural Characterization of GO and GO/EP Composite

**Figure 1:**
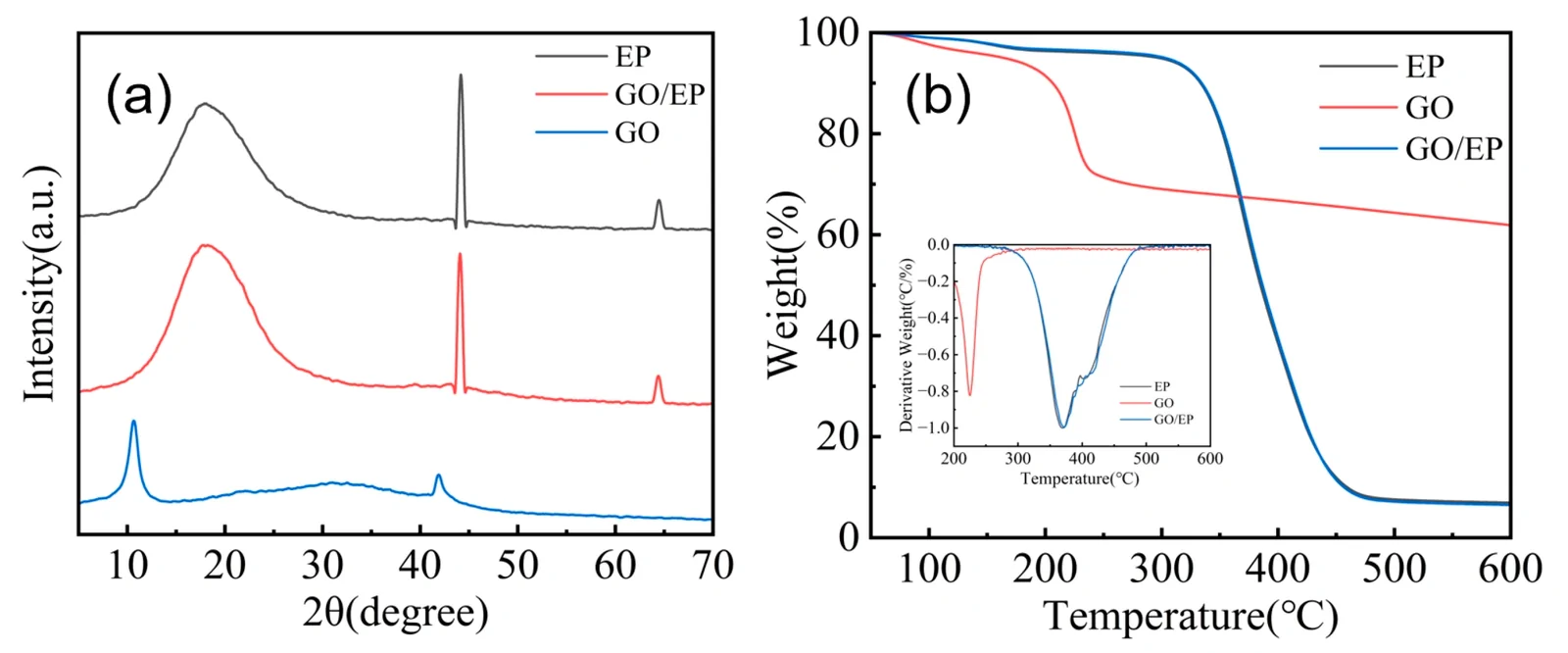
XRD patterns of (**a**) GO powders, EP and GO/EP coated steel samples; (**b**) TGA curves of GO powders, EP and GO/EP composites.

**Figure 2:**
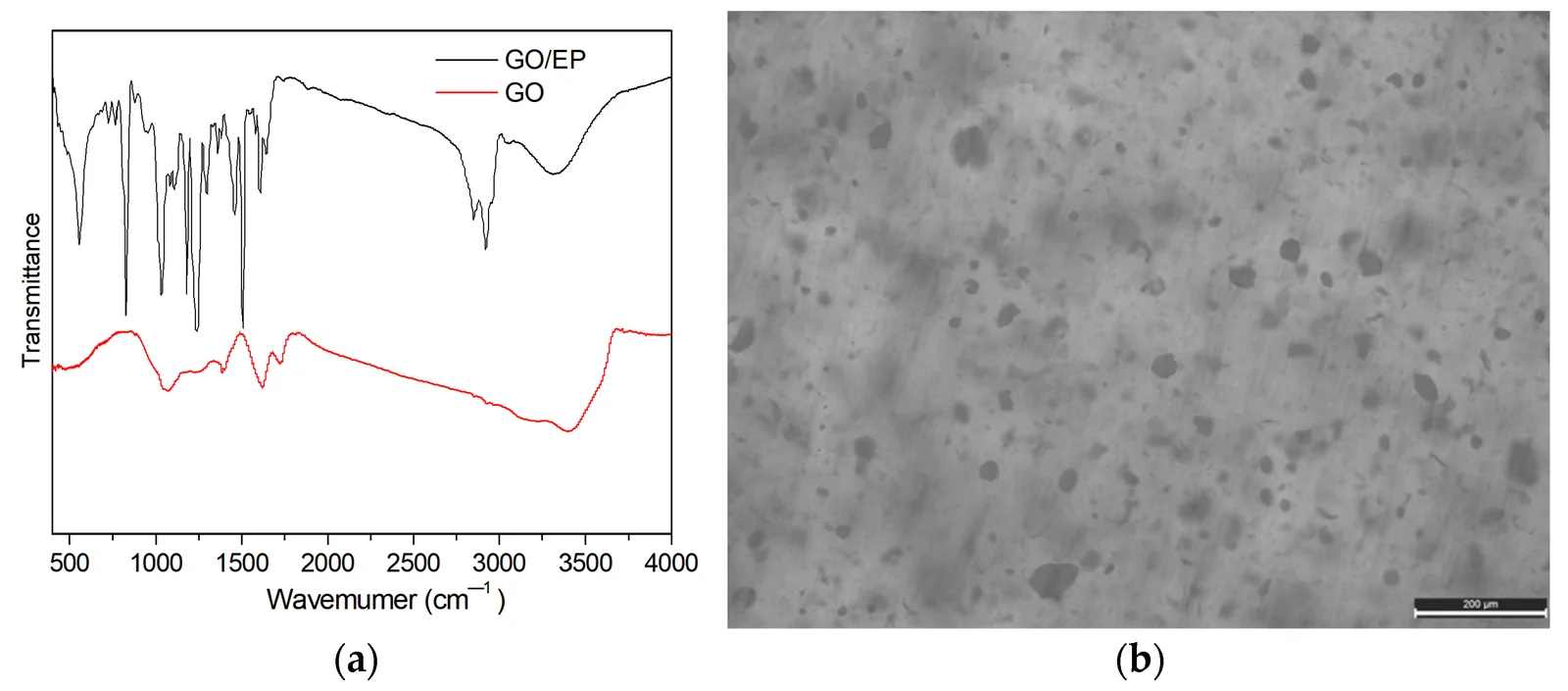
(**a**) FTIR spectra of GO and GO/EP; (**b**) optical image of GO/EP composite.

As shown in Figure 1, GO exhibits a characteristic (002) diffraction peak at 2θ ≈ 10.5°, confirming its multilayered structure [23]. In the GO/EP composite, this characteristic peak of GO is completely absent, while the broad peak around 18.5° originates from the amorphous structure of the epoxy matrix. The sharp peaks around 44°and 64°are ascribed to the (110) and (220) crystal planes of the carbon steel substrate. The disappearance of GO’s characteristic peak can be partially attributed to the low content of GO in the composite, as well as its uniform distribution in the epoxy matrix, which suppresses its crystallization behavior. As shown in Figure 1b, GO has much lower thermal stability than neat EP, with its maximum thermal decomposition occurring around 220 °C. Its TGA curve seems different from the one reported previously [25]. It is plausible that this may originate from the different production batches of the GO product, as well as from the gradual evolution of GO’s properties during storage. Notably, even with only 0.1 wt% GO incorporated, the maximum weight-loss temperature of the GO/EP composite shifts from 370 °C to 375 °C, indicating enhanced overall thermal stability despite the inferior thermal stability of GO itself. This improvement can be attributed to the uniform distribution of GO nanosheets in the matrix and the interfacial interactions between GO and the polyamide-epoxy curing system. As shown in Figure 2a, the FTIR spectrum of GO displays a broad -OH stretching vibration peak at 3412 cm^−1^. In the spectrum of GO/EP, this peak shifts to a lower wavenumber (3320 cm^−1^), which demonstrates the existence of interactions between GO and the epoxy resin matrix. In fact, previous work has confirmed that strong interactions exist between the curing agent polyamide 651 even at 50 °C [34], which is lower than the 60 °C curing temperature used for GO/EP in this work. These interactions between the filler and the resin system promote the uniform distribution of GO sheets in the epoxy matrix, which is confirmed by the corresponding optical image (Figure 2b). The image shows a typical uniform dispersion of GO sheets in the resin, consistent with results reported in recent studies [37].

### 3.2. Corrosion Resistance of Epoxy Coatings

#### GO/EP Coatings

**Figure 3:**
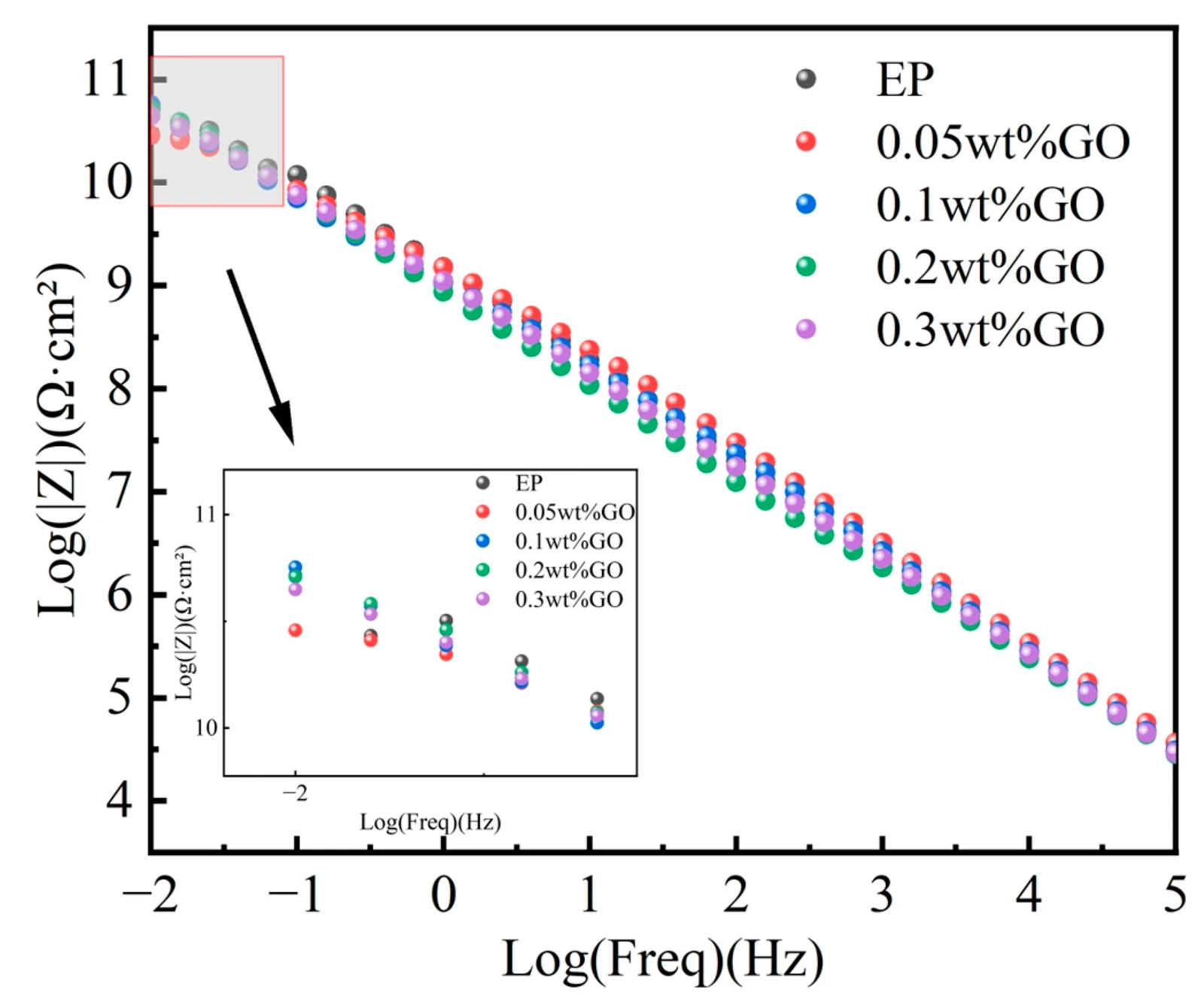
The EIS Bode plots of neat EP and GO/EP coatings modified with different GO content in 3.5 wt% NaCl solution.

The low-frequency impedance modulus (|Z|_0.01 Hz_) is widely recognized as an indicator for evaluating the overall anticorrosion performance of organic coatings [25,38]. Figure 3 shows the EIS Bode plots of GO/EP coatings modified with different GO contents in 3.5 wt.% NaCl solution. It is clear that a low GO content of 0.05 wt.% is unfavorable, and even weakens the corrosion inhibition performance of the coating, as its |Z|_0.01 Hz_ ((2.88 × 10^10^ Ω·cm^2^) is lower than that of the neat EP coating (5.13 × 10^10^ Ω·cm^2^). However, when 0.1 wt% GO is incorporated into the EP coating, its |Z|_0.01 Hz_ increases to 5.66 × 10^10^ Ω·cm^2^, which is the highest among all tested coatings. Further increasing the GO content to 0.3 wt.% leads to a general decrease in |Z|_0.01 Hz_. These test results confirm that the corrosion resistance of the composite coating depends on its GO content, and the best-performing concentration of GO is identified as 0.1 wt%, which is consistent with a recent report [37]. In the following sections, the epoxy composite coating containing 0.1 wt% GO is labeled as ‘GO/EP’.

### 3.3. Long-Term Anticorrosion Performance of Composite Coatings

**Figure 4:**
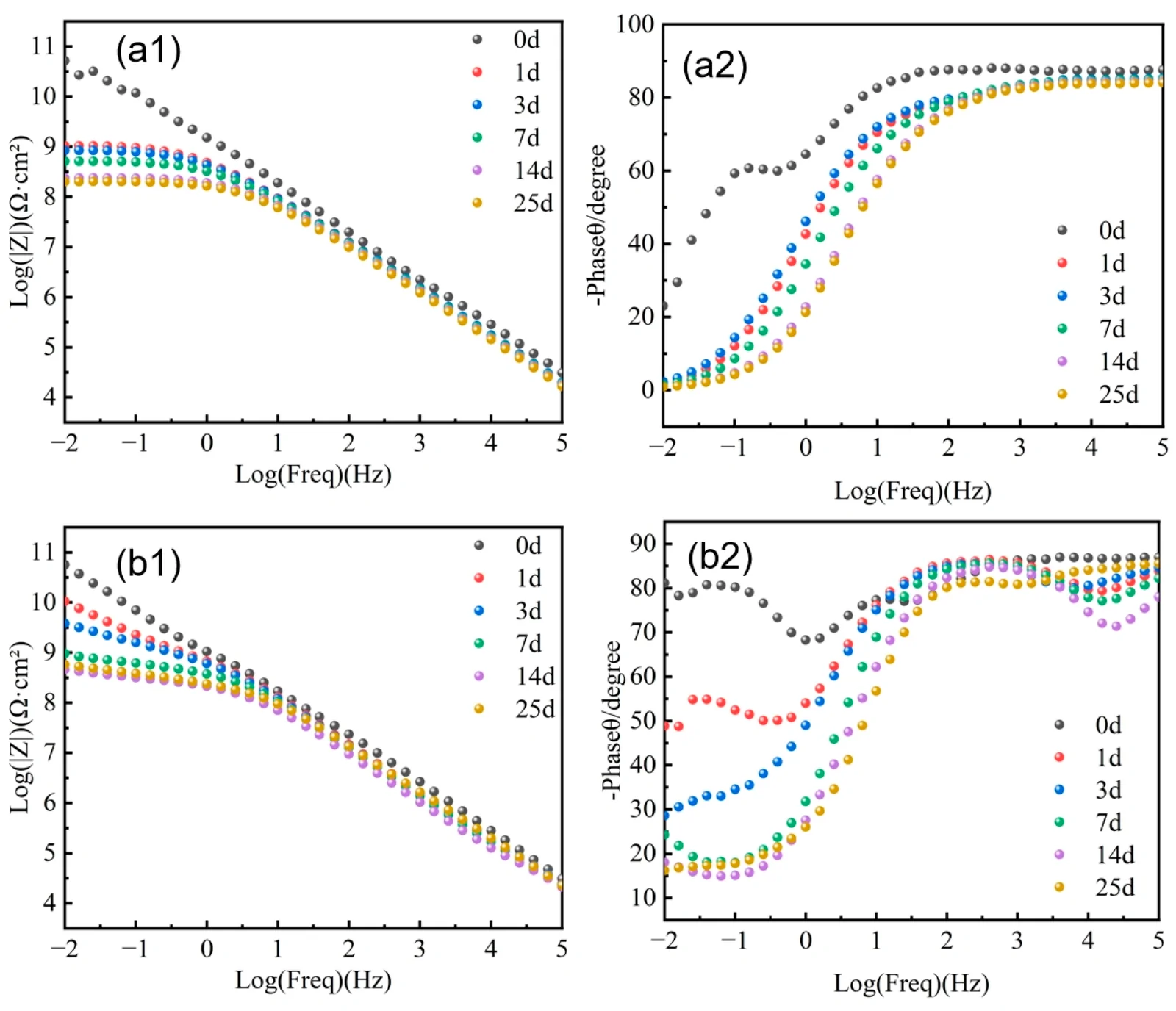
Bode plots of the coatings after different immersion times: (**a1**,**a2**) EP coating; (**b1**,**b2**) GO/EP coating.

To evaluate the long-term corrosion resistance of the composite coating, a continuous immersion test was conducted. The evolution of the protective performance of the epoxy coatings during up to 25 days of immersion in 3.5 wt.% NaCl solution was monitored via EIS analysis (Figure 4 and Figure S1). As revealed by the Bode plots in Figure 4, both neat EP and GO/EP coatings exhibited high |Z|_0.01 Hz_ values at the initial immersion stage (0 d), along with broad high-phase-angle plateaus spanning a wide frequency range, which is a typical characteristic of a purely capacitive response. This indicates that the coatings were dense and structurally intact, with negligible ingress of corrosive species, demonstrating a clear physical barrier effect. As immersion time increased, the |Z|_0.01 Hz_ of both coatings declined continuously, as corrosive species including Cl^−^ and H_2_O gradually permeated through the inherent micropores and defects inside the coatings. This process could be accompanied by a progressive shrinkage of the high-phase-angle plateau toward lower frequencies, which corresponds to the continuous degradation of the coating barrier performance.

**Figure 5:**
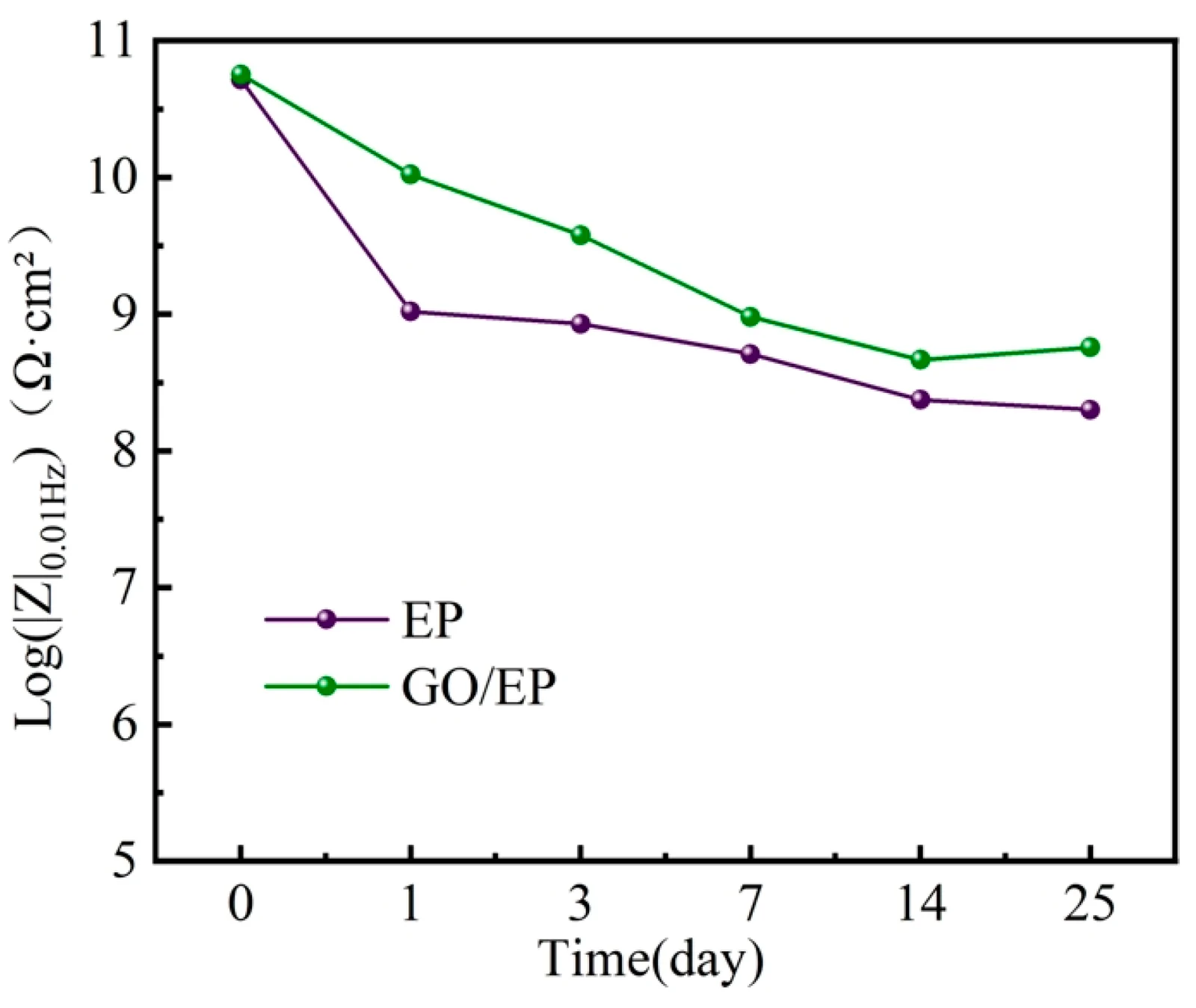
The dependence of the impedance for EP and GO/EP coatings on the immersion time in 3.5 wt.% NaCl solution.

Figure 5 presents a comparison of |Z|_0.01 Hz_ evolution for the two coating systems over the 25-day immersion period. Clearly, both coatings exhibited high |Z|_0.01 Hz_ values at the initial immersion stage. Their impedance values decreased gradually with immersion time, caused by the continuous infiltration of corrosive species from the NaCl solution into the coating. It can be observed that the |Z|_0.01 Hz_ of neat EP decreased much faster than that of GO/EP sample within the first day of immersion, indicating a more rapid degradation of its physical barrier function. In contrast, GO/EP coating showed a significantly slower decay rate, which demonstrates that GO provides a strong barrier effect for the epoxy coating. Throughout the entire immersion period, the |Z|_0.01 Hz_ of neat EP coating remained lower than that of GO/EP coating, which confirms that GO nanosheets positively improve the corrosion resistance of epoxy coatings.

**Figure 6:**
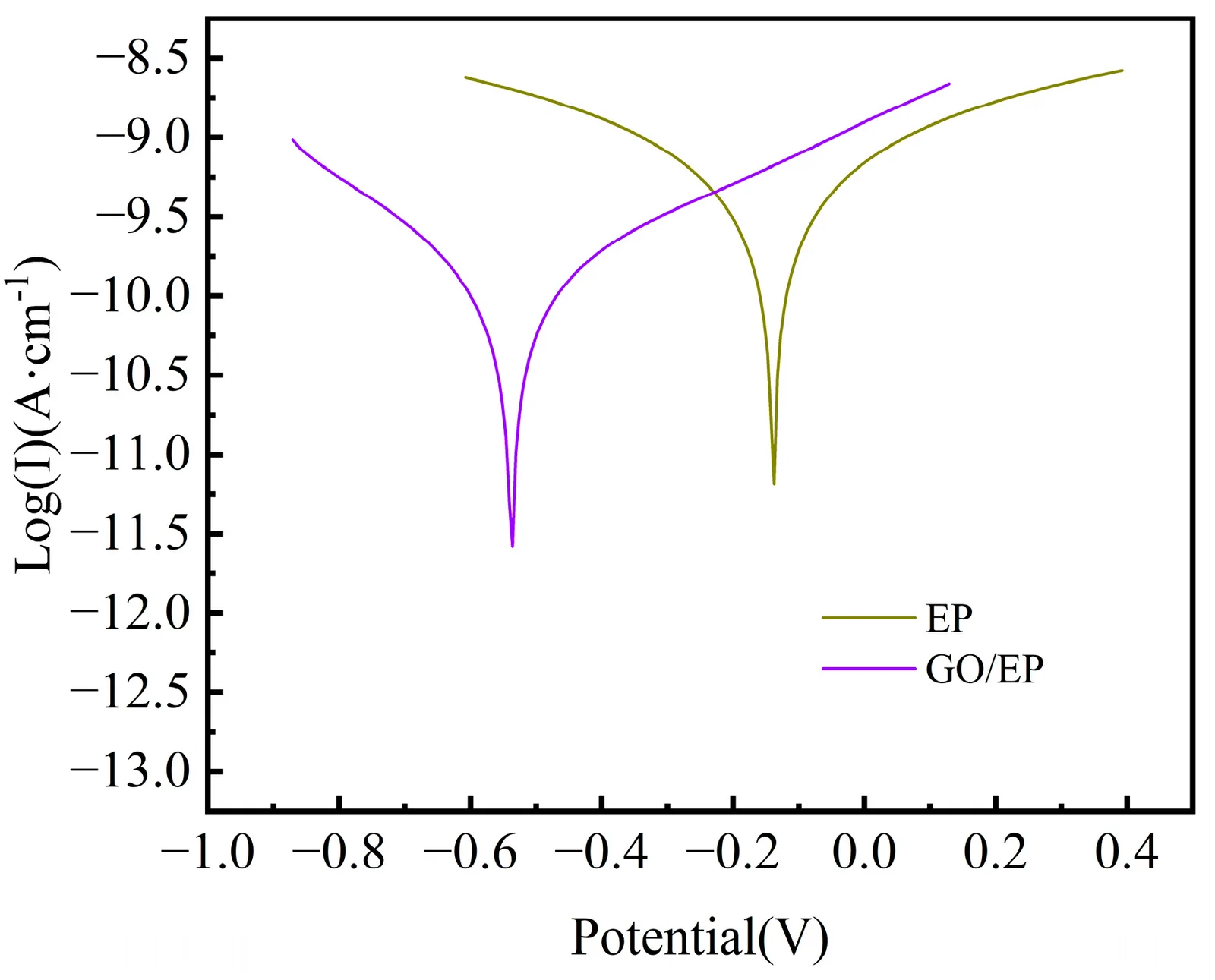
Potentiodynamic polarization curves of EP and GO/EP coatings after 25 d immersion in 3.5 wt.% NaCl solution.

Furthermore, after 25 days of immersion, GO/EP coating showed a much lower corrosion current density (I_corr_) than neat EP, as illustrated by the Tafel curve in Figure 6. The I_corr_ of GO/EP was 4.69 × 10^−11^ A cm^−2^, only approximately one-third that of neat EP (1.57 × 10^−10^ A cm^−2^). The significantly reduced I_corr_ further confirms that incorporating GO effectively improves the corrosion resistance of epoxy coatings.

**Figure 7:**
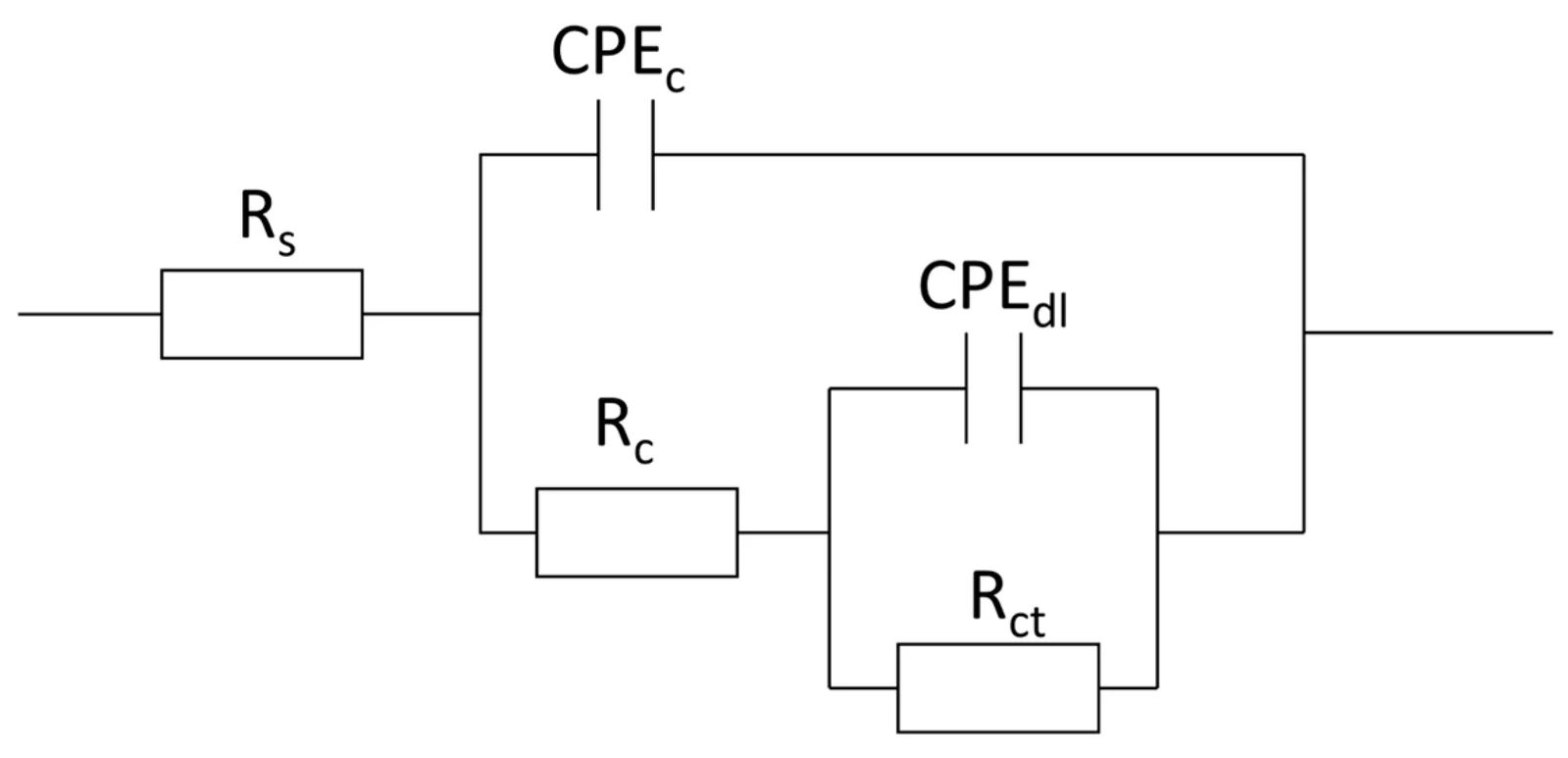
Equivalent circuit for electrochemical impedance fitting.

To clarify the mechanism behind the different anticorrosion behaviors of the coatings during immersion, the EIS data were fitted using an equivalent circuit, which is presented in Figure 7. In the equivalent circuit, a constant phase element (CPE) was used in place of an ideal capacitor to improve fitting accuracy. In this circuit, Rs denotes the solution resistance; Rc and CPEc correspond to coating resistance and coating capacitance, respectively; and Rct and CPEdl reflect the interfacial charge transfer process and double-layer behavior. The key parameters obtained from the fitting are summarized in Table 1. Rc directly reflects the resistance of the coating to corrosive medium penetration and acts as a core indicator of its physical barrier performance [39,40]. At the initial immersion stage (day 1), the Rc of GO/EP coating reached 9.06 × 10^8^ Ω·cm^2^, which was significantly higher than that of the neat EP coating, confirming that the incorporation of GO effectively improved the compactness of the coating. As immersion time prolonged, electrolyte continuously penetrated through the internal defects of the coatings, leading to a declining trend in the Rc of both coatings. Compared with GO/EP, neat EP coating experienced more severe deterioration: its Rc dropped by more than one order of magnitude after the immersion test, indicating a substantial decline in barrier performance. In contrast, the attenuation rate of Rc for the GO/EP coating was much slower, demonstrating that the degradation of its barrier performance was significantly retarded. The evolution of Rct was consistent with that of Rc. The Rct values of both coatings decreased gradually as immersion time increased, and GO/EP coating consistently maintained Rct values larger than that of EP coating. This indicates that GO/EP coating has superior resistance to interfacial corrosion reactions and a prominent inhibition effect on corrosion progression. While the CPEc of both coatings showed insignificant change during immersion, the CPEdl exhibited an increasing trend with prolonged immersion, which corresponds to the continuous expansion of the interfacial double-layer region as corrosion progresses. The fitting results indicate that GO nanosheets enhance the physical barrier performance of the coating and retard the penetration of corrosive media, endowing the coating with both excellent initial barrier capability and long-term anti-corrosion performance, and effectively extending the service life of the epoxy coating.

### 3.4. Thermal Cycling Resistance of Coatings

**Figure 8:**
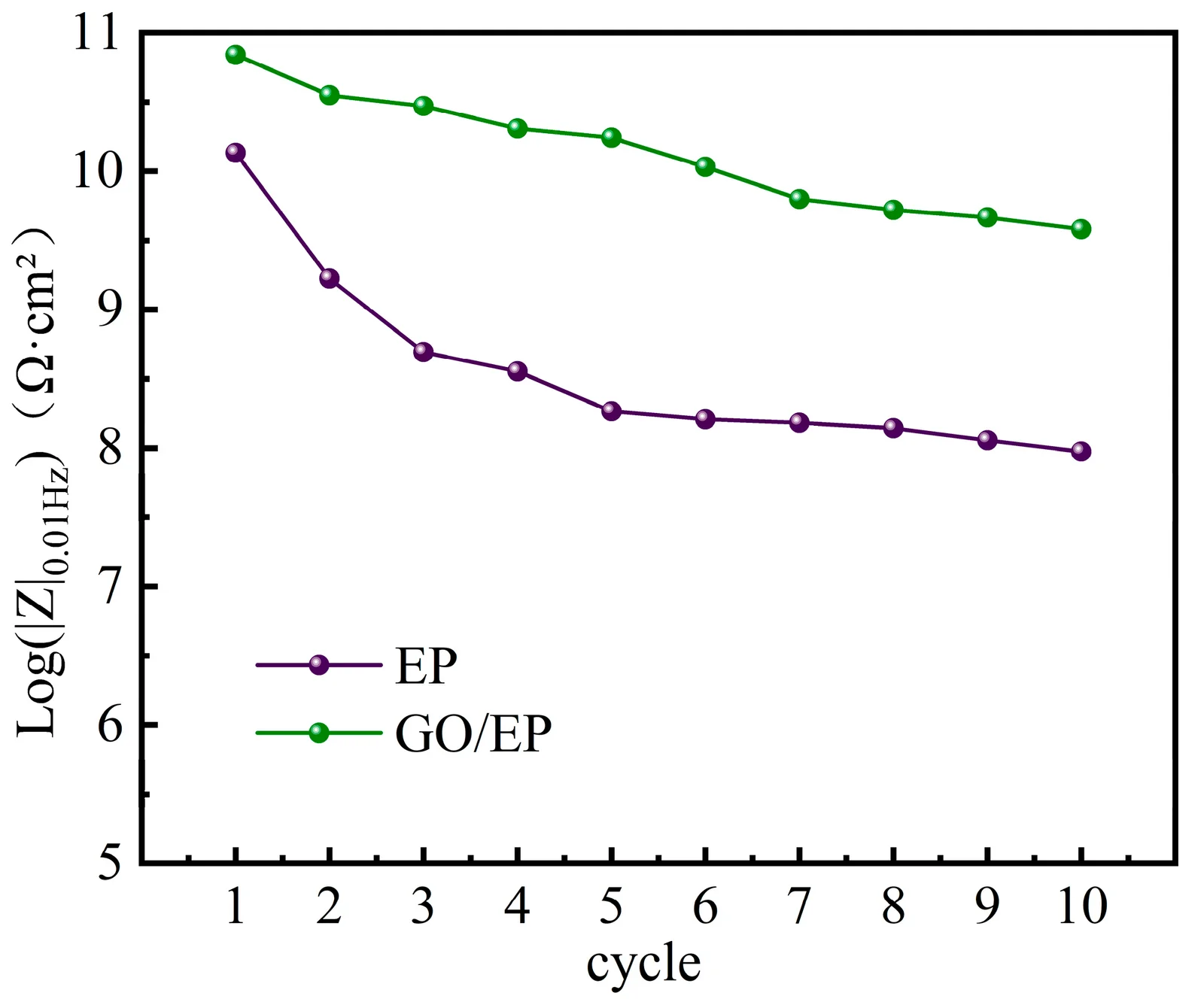
Variation in low-frequency impedance modulus (|Z|_0_._01 Hz_) of EP and GO/EP coatings with thermal cycling cycle.

Thermal cycling tests were conducted to evaluate the service stability of the coatings under alternating temperature conditions. Each cycle consisted of 12 h at −30 °C followed by 12 h at 60 °C. Figure 8 presents the evolution of |Z|_0.01 Hz_ for both EP and GO/EP coating systems across different numbers of cycles. The impedance of both neat EP and GO/EP coatings decreased continuously as the number of cycles increased. For neat EP coating, the impedance dropped sharply within the first 5 cycles, after which the degradation rate slowed down. In contrast, the GO/EP coating maintained higher impedance values than neat EP coating throughout the entire thermal cycling test, retaining satisfactory anti-corrosion performance.

Currently, the mechanism for the effect of incorporating GO on enhancing the thermal cycling resistance of the coating is unavailable yet, but may be related to the reinforcement effect and the ‘maze effect’ of GO nanosheets in the polymer matrix.

### 3.5. UV Aging Resistance of Composite Coatings

#### 3.5.1. Corrosion Resistance

**Figure 9:**
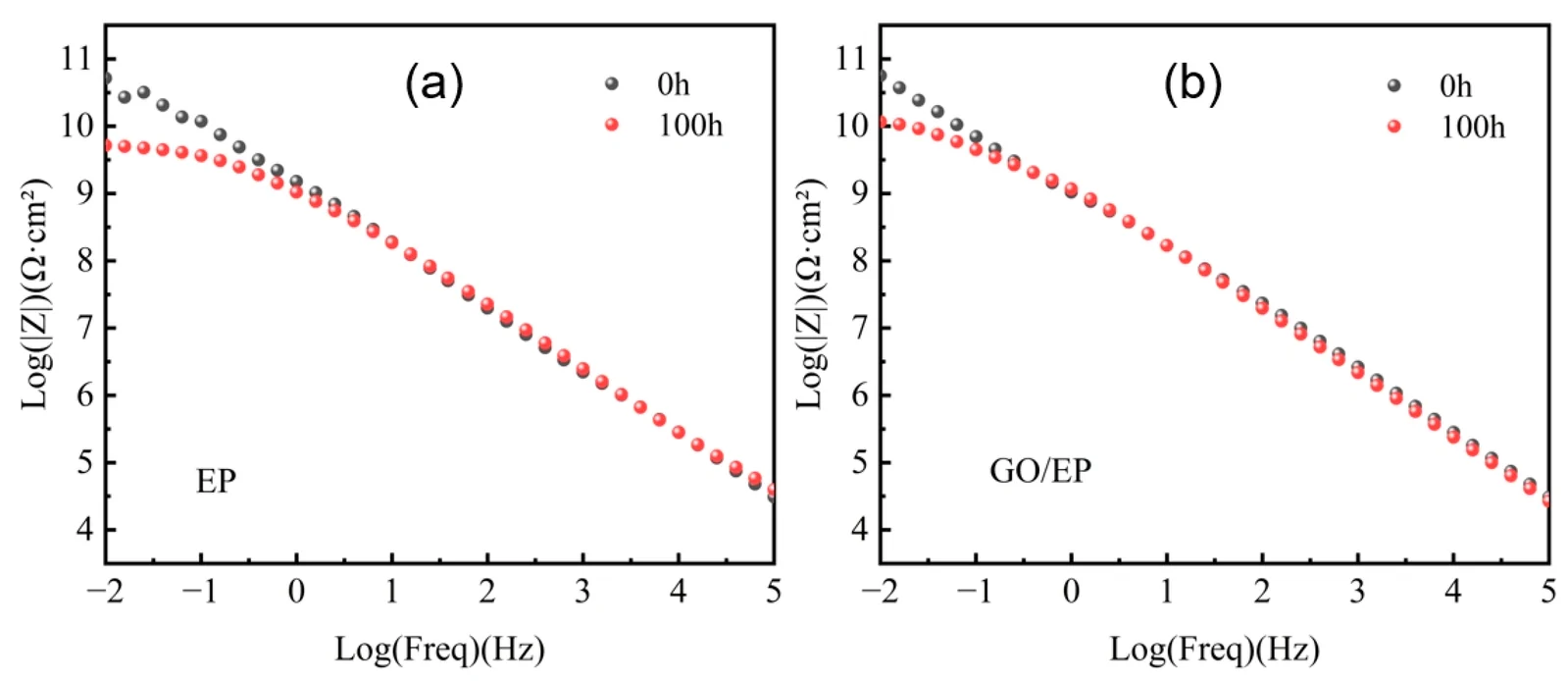
EIS Bode plots of the coatings before and after UV aging test: (**a**) EP coating; (**b**) GO/EP coating.

An accelerated UV aging test was conducted to evaluate the effect of photo-oxidation on the anticorrosion performance of both EP and GO/EP coating systems. The EIS Bode plots obtained before and after aging, as well as the corresponding fitting parameters derived from the equivalent circuit model, are presented in Figure 9 and Table 2, respectively. The corresponding Nyquist plots were also given as Figure S2 in the Supplementary Materials. After 100 h of UV aging, the |Z|_0.01 Hz_ values of both coatings decreased, along with reductions in both Rc and Rct, alongside increases in the constant phase elements of the coating (CPEc) and the double layer (CPEdl).

As shown in Table 2, neat EP coating had initial values of R_c_ = 4.64 × 10^9^ Ω·cm^2^ and R_ct_ = 4.06 × 10^10^ Ω·cm^2^. After the UV aging test, these values decreased to 2.11 × 10^9^ Ω·cm^2^ and 1.15 × 10^10^ Ω·cm^2^, respectively, meaning both parameters dropped by more than 50%, alongside a significant increase in CPE_c_ and CPE_dl_. Compared to neat EP coating, GO/EP coating maintained a higher level of protective performance after UV aging. Its post-aging Rc and Rct values were 2.21 × 10^9^ Ω·cm^2^ and 1.26 × 10^10^ Ω·cm^2^, respectively, and the magnitude reduction of |Z|_0.01 Hz_ (0.68 orders of magnitude) was smaller than that of neat EP coating (one order of magnitude). It is reasonable to attribute the larger corrosion resistance of the GO/EP coating after UV aging to the dual functions of GO nanosheets. First, GO nanosheets can reflect and absorb part of the incident UV radiation, reducing the UV energy reaching the epoxy resin matrix and slowing down the photo-oxidative degradation process. Second, GO nanosheets may form a tortuous diffusion path inside the coating, which hinders the permeation of corrosive media and mitigates the degradation of barrier performance caused by UV aging.

#### 3.5.2. Gloss and Surface Properties

**Figure 10:**
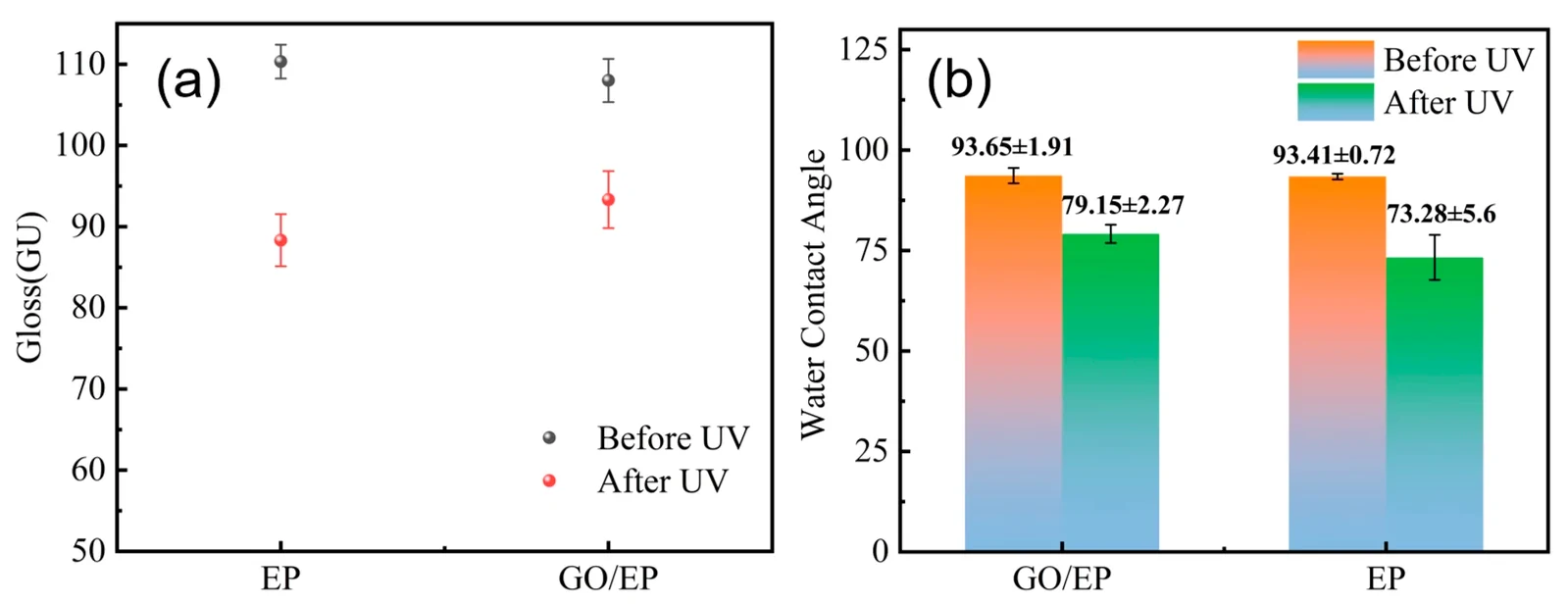
(**a**) Gloss; (**b**) water contact angle of EP and GO/EP coatings before and after UV aging.

Figure 10 shows the changes in gloss and surface hydrophobicity of EP and GO/EP coatings before and after UV aging. Before UV exposure, both coatings had relatively high initial gloss values, with no significant difference between them (Figure 10a). After UV aging, the gloss of both coatings decreased to varying degrees. The neat EP coating saw a more pronounced drop: from approximately 110 GU to around 88 GU, representing a 20.0% reduction. In comparison, the gloss of GO/EP coating decreased from approximately 108 GU to around 93 GU, with a much smaller reduction of 13.9%. This result aligns with the gloss evolution trend observed in epoxy coatings reinforced with functionalized GO@CNT hybrids reported recently [40].

Figure 10b shows the variation in water contact angle (WCA) of the two coatings before and after UV aging. Before UV aging, both EP and GO/EP coatings exhibited good initial hydrophobicity, with WCA values exceeding 93° (93.41° and 93.65°, respectively) and no significant difference between them. After UV irradiation, the WCA of both coatings decreased to varying degrees. This indicates that UV radiation either destroyed the chemical structure of the coating surface or generated micro-defects, ultimately reducing the surface hydrophobicity of the coatings. Notably, neat EP coating showed a more evident WCA reduction, dropping from 93.41° to 73.28°, while the WCA of the GO/EP coating only decreased to 79.15°, proving that this composite coating retains better hydrophobic performance after aging.

**Figure 11:**
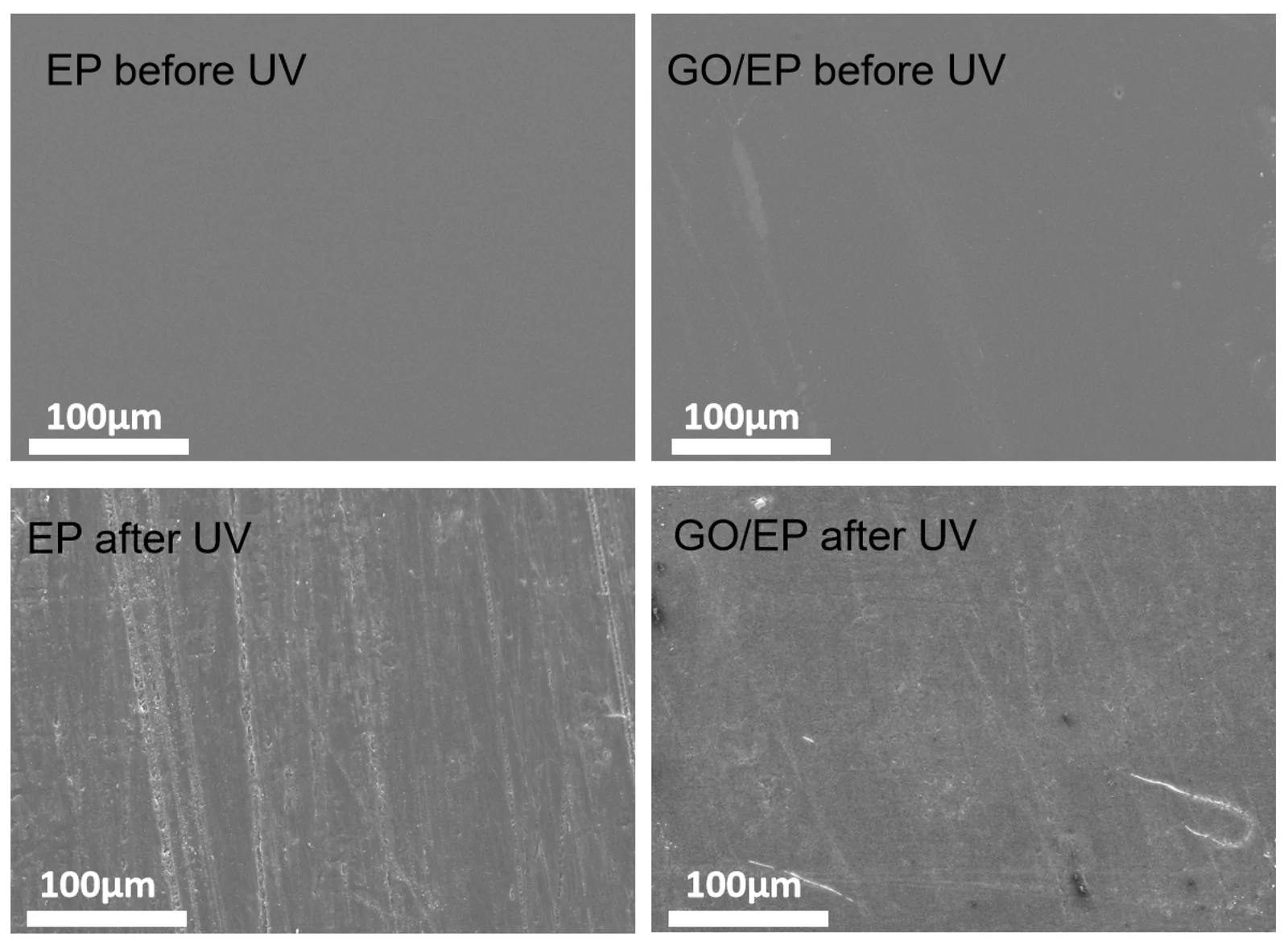
SEM image of the surface of EP and GO/EP coating before and after UV aging test.

The SEM image of the coating surface (Figure 11) corroborates the trends observed in gloss and water contact angle. Before UV aging, both coatings had relatively smooth, compact surfaces free of obvious defects. After UV aging, the surface of neat EP coating displayed numerous dense parallel cracks and pronounced groove-like damage, confirming that UV radiation caused severe surface degradation of the epoxy matrix. In comparison, although minor surface damage was still visible on the GO/EP coating, both the number and depth of cracks were reduced. The surface roughness of both coatings before and after UV aging test were shown as Figure S3 in the Supplementary Materials. Evidently, the neat EP coating showed a greater increase in roughness (rising from 0.075 to 0.132), while the roughness of the GO/EP coating only increased from 0.090 to 0.112, confirming that the composite coating has superior UV aging resistance compared to the neat EP coating. These results demonstrate that incorporating GO into the epoxy coating improves its resistance to UV irradiation, effectively slowing the photo-oxidative degradation process on the coating surface.

#### 3.5.3. Adhesion to Steel Substrate

**Figure 12:**
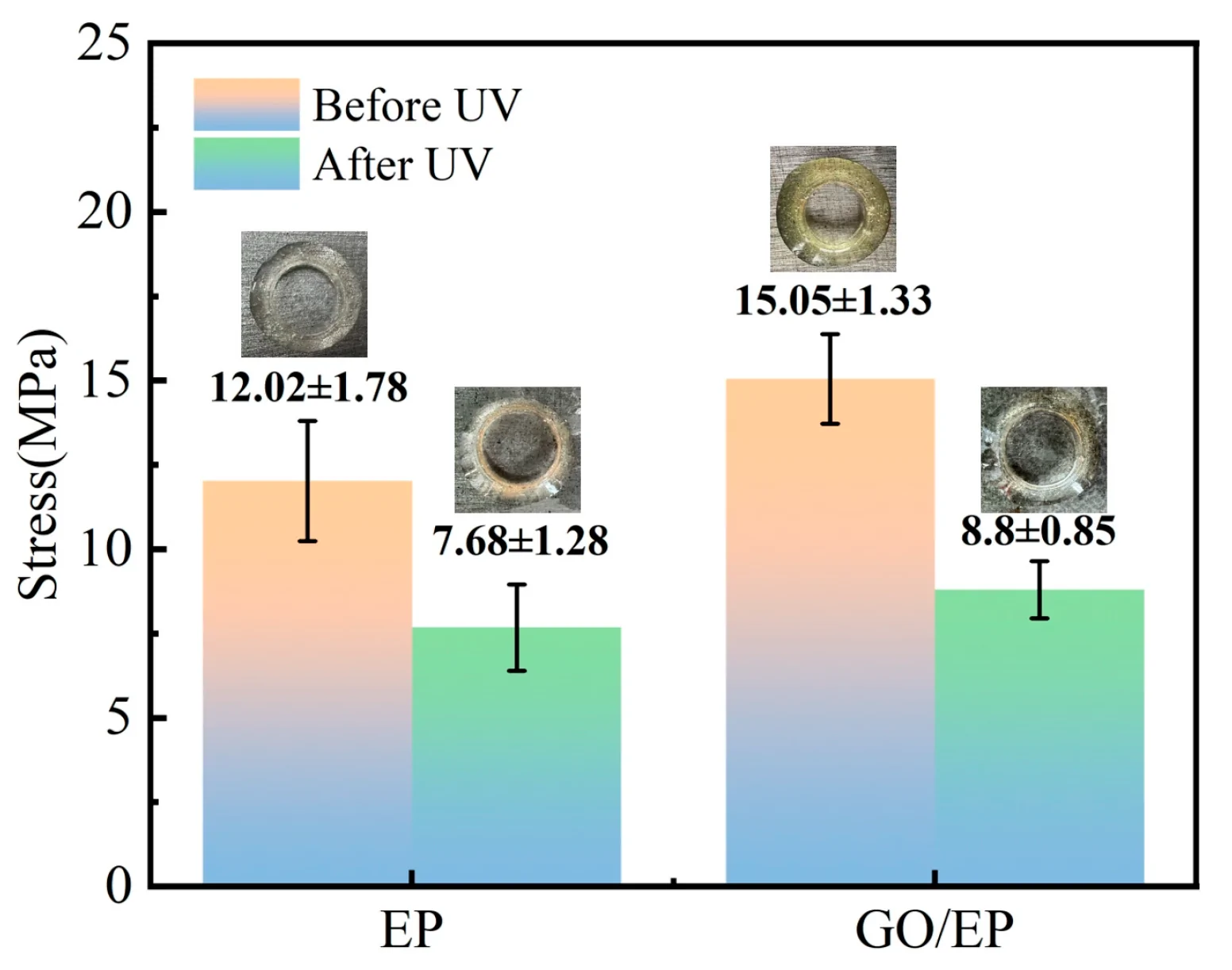
The adhesion strength of EP and GO/EP coating to steel substrate before and after UV aging.

The changes in adhesion strength further illustrate the extent of UV-induced damage at the coating–steel substrate interface (Figure 12). Before UV aging, the adhesion strengths of EP and GO/EP coatings were 12.02 ± 1.78 MPa and 15.05 ± 1.38 MPa, respectively, confirming that incorporating GO into EP improves the coating’s adhesion to the steel substrate. This improvement can be attributed to the enhanced interfacial interactions between the reactive functional groups on GO and the epoxy matrix (Figure 2a), which endows GO with a strong reinforcing effect on the epoxy matrix. After UV aging, the adhesion strength of both coatings decreased significantly. Neat EP coating dropped from 12.02 ± 1.78 MPa to 7.68 ± 1.28 MPa, with a retention rate of 63.9%, while GO/EP coating decreased from 15.05 ± 1.38 MPa to 8.80 ± 0.85 MPa, with a retention rate of 58.5%. Moreover, as shown in the inset photos of typical samples after the test, portions of both the EP and GO/EP coating layers remained on the steel substrate, indicating that the coatings underwent cohesive failure. It is clear that even after UV aging, the adhesion strength of the GO/EP coating remains higher than that of the neat EP coating, which further proves that GO improves the UV aging resistance of the coating from the perspective of the bonding between the coating and the steel substrate.

#### 3.5.4. Water Uptake Behavior

**Figure 13:**
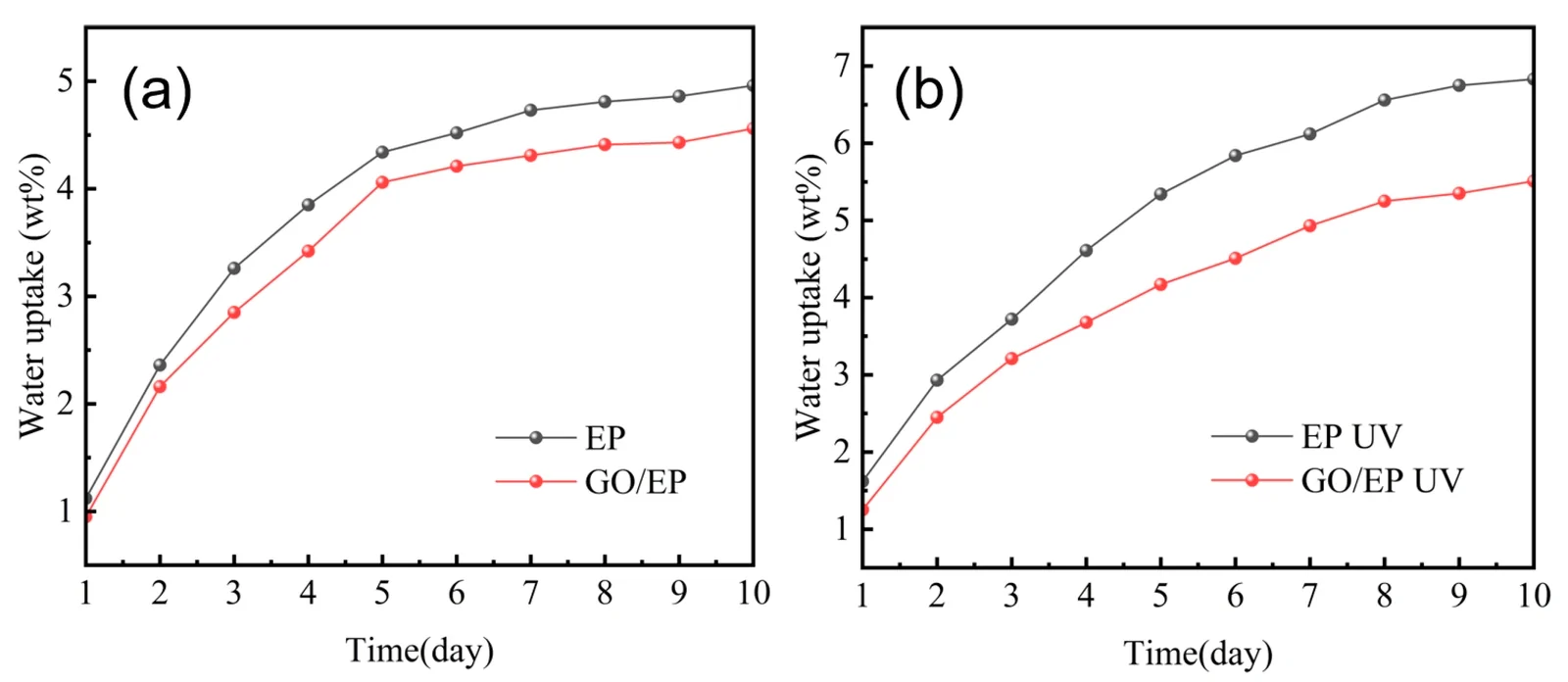
Water uptake behaviro of EP and GO/EP coatings (**a**) before and (**b**) after UV aging.

Water permeability is a critical parameter for evaluating the long-term service stability of anticorrosion coatings in harsh outdoor environments [41,42]. The water uptake tests of the coating materials were performed according to ISO 2812-2:2018 [43]. Figure 13a,b compare the water uptake behavior of the two coatings as a function of immersion time, before and after UV aging. Before aging, the neat EP coating had a saturated water uptake of 4.86 wt.%, while incorporating GO reduced the saturated water uptake to 4.56 wt.%. This can be attributed to the “maze effect” of GO in the epoxy matrix, which greatly extends the diffusion pathway of water molecules. After UV aging, the water uptake of the GO/EP coating reached 5.51 wt.%, representing an increase of only 0.95%, which is about half the increment measured for neat EP (an increase of 1.87%). After UV aging, the inevitable formation of micro-cracks and increased free volume on the surface and interior of the coating created more accessible permeation channels for water molecules, leading to a significant increase in water uptake for both coatings (Figure 13). The lower water uptake of GO/EP confirms that even when the epoxy matrix is partially damaged by UV aging, the two-dimensional barrier structure of GO can still effectively maintain the compactness of the coating interior. It significantly inhibits the permeation of corrosive water media toward the coating–steel interface, thus endowing the coating with higher anticorrosion performance after UV aging (Figure 9).

### 3.6. Wear Resistance and Tribological Performance

**Figure 14:**
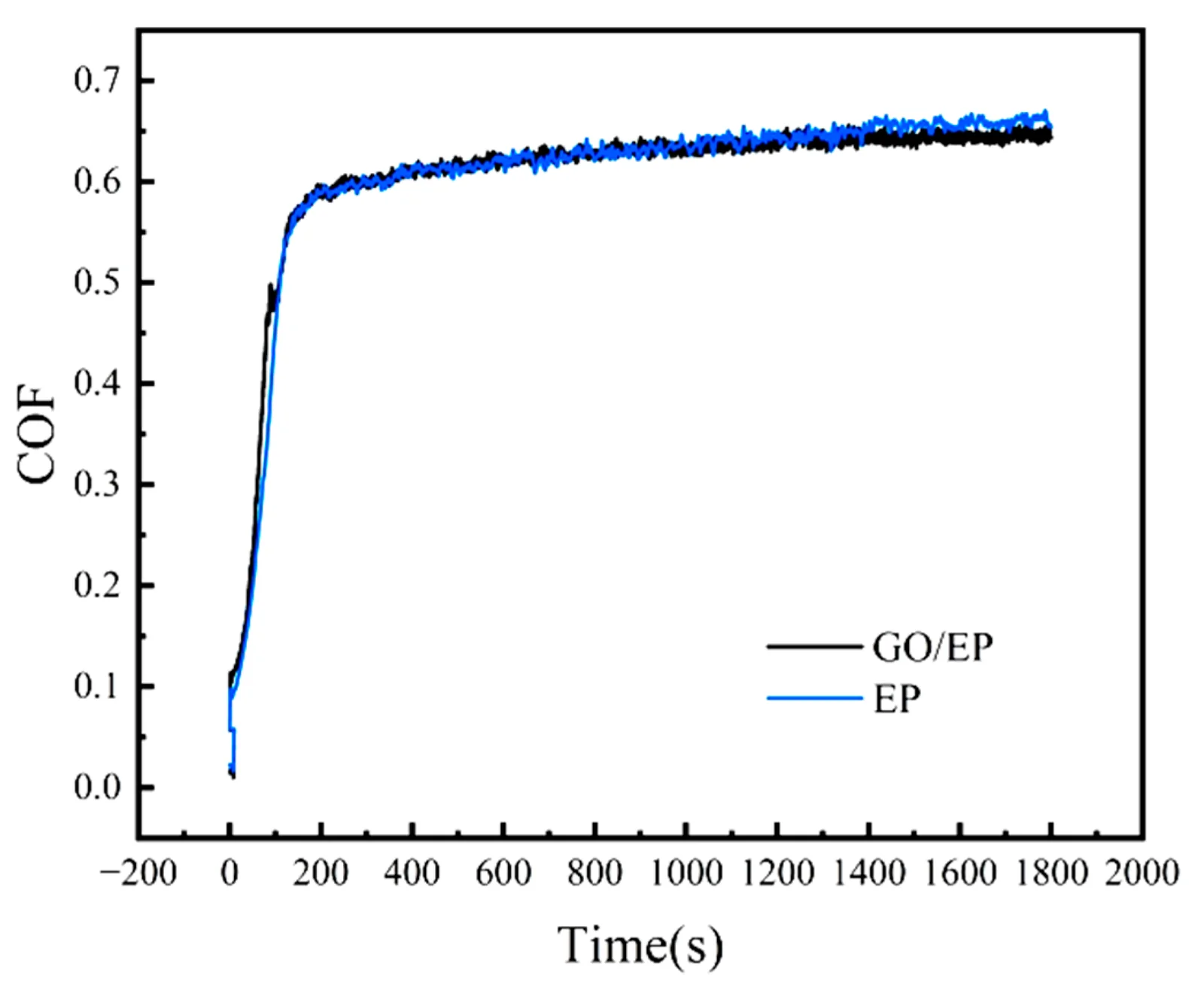
Variation curves of friction coefficient of EP and GO/EP coatings with testing time.

Besides corrosion resistance, the wear resistance of the coatings is also critical to their service life [44]. Therefore, tribological tests were conducted on both EP and GO/EP coatings. As shown in Figure 14, the entire friction process of both coatings can be divided into two stages: an initial running-in stage (approximately 200 s) and a subsequent steady wear stage. In the initial stage, the friction counterpart gradually comes into contact and interacts with the micro-asperities on the coating surface, leading to a continuous increase in the real contact area, which is accompanied by a rapid rise in the friction coefficient. After this stage, the friction system enters the stable wear stage, which is characterized by a significantly reduced fluctuation amplitude of the friction coefficient that generally remains at a steady level. In terms of the average friction coefficient, GO/EP coating exhibits a coefficient of friction (COF) of 0.61, which is almost identical to that of neat EP.

**Figure 15:**
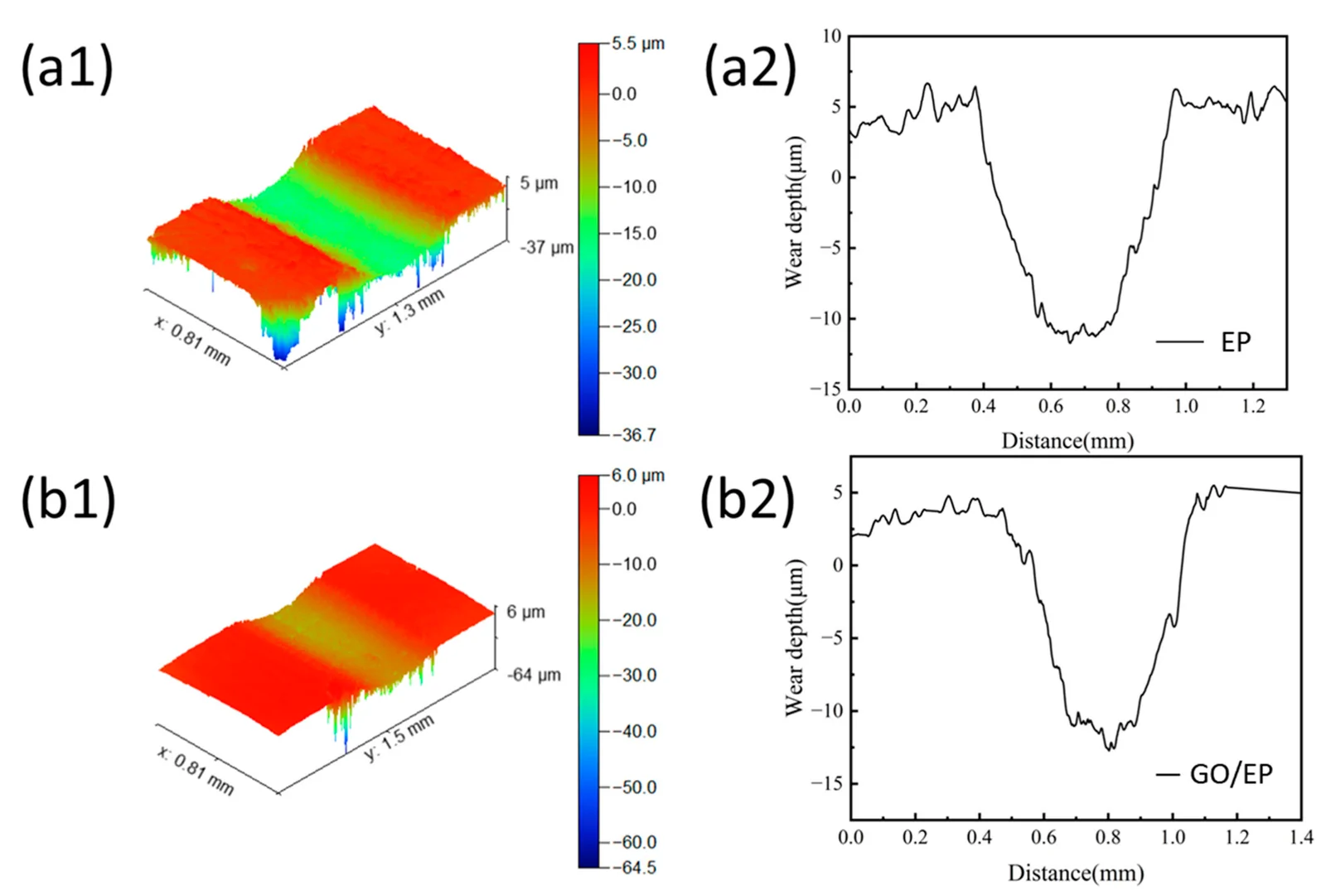
3D morphologies and cross-section profiles of wear tracks on the three coatings. (**a1**,**a2**) EP coating; (**b1**,**b2**) GO/EP coating.

The wear rate is the core quantitative parameter for evaluating the wear resistance of coatings [45]. To visually characterize the degree of wear damage to the coatings, three-dimensional (3D) profilometry was used to analyze the cross-section profiles of the wear tracks, and the results are presented in Figure 15. The neat EP coating had a wide, deep wear track with obvious undulations in its cross-section profile, indicating that severe adhesive peeling and plastic deformation occurred during the friction process. Quantitative analysis of the cross-section profiles in Figure 15(a1,a2) shows that the wear track has a width of 0.601 mm and a maximum wear depth of 16.04 μm, confirming more severe wear damage. After incorporating GO, the wear track depth of the composite coating decreased and material peeling was alleviated. GO/EP composite coating has a wear track width of 0.591 mm, which is comparable to that of neat EP coating, while the maximum wear depth drops to 14.99 μm. This indicates that incorporating GO into EP coating mainly reduces vertical material removal through friction stress dissipation and self-lubricating effects, while its inhibitory effect on the lateral expansion of the wear track is relatively limited. Correspondingly, the calculated wear rate of neat EP coating is 3.34 × 10^−15^ m^3^/N·m. By contrast, the GO/EP composite coating has a lower wear rate of 2.41 × 10^−15^ m^3^/N·m, which is approximately 28% lower than that of neat EP coating. This can be ascribed to the role of two-dimensional GO nanosheets as reinforcing and toughening agents in the resin matrix, which effectively dissipate contact stress during friction. However, the real tribological mechanism still requires more in-depth investigation, and further research on this topic will be conducted in future work.

## 4. Conclusions

In this study, GO was used as a multifunctional filler to prepare a modified epoxy composite coating. Its regulatory effects on the overall performance of the coating were systematically investigated, including anticorrosion performance, thermal cycling resistance, UV resistance, wear resistance, and adhesion strength to metal substrates. The results are summarized as follows:

(1) With the addition of only 0.1 wt% GO, the resulting GO/EP composite coating exhibits higher adhesion strength (15.08 MPa) than the neat epoxy coating (12.02 MPa), along with lower wear rate and water uptake.
(2) The modified coating also displays excellent corrosion resistance: after long-term immersion in a NaCl solution for 25 days, it has a corrosion current density of 4.69 × 10^−11^ A·cm^−2^, which is approximately 1/3 that of the neat epoxy coating.
(3) After thermal cycling testing, the impedance of GO/EP coating remains ~40 times higher than that of neat epoxy coating.
(4) By incorporating 0.1 wt% GO, the wear rate of the epoxy coating was reduced by approximately 30%.

This study demonstrates that GO nanoadditives can improve multiple service performances of epoxy coatings, providing a feasible and promising strategy for developing long-term protective coatings for outdoor applications.

## Figures and Tables

**Table 1:** EIS fitting parameters of different coatings after different immersion duration.

Coating	Time (day)	Rc (Ω·cm^2^)	Rct (Ω·cm^2^)	CPEc (S·sn·cm^−2^)	CPEdl (S·sn·cm^−2^)
EP	0	4.64 × 10^9^	4.06 × 10^10^	4.75 × 10^−11^	9.23 × 10^−11^
	1	3.71 × 10^8^	4.88 × 10^8^	2.10 × 10^−10^	5.87 × 10^−10^
	3	2.04 × 10^8^	8.80 × 10^8^	1.86 × 10^−10^	3.27 × 10^−10^
	7	9.61 × 10^7^	4.33 × 10^8^	1.87 × 10^−10^	4.33 × 10^−10^
	14	3.92 × 10^7^	2.04 × 10^8^	1.92 × 10^−10^	5.44 × 10^−10^
	25	1.81 × 10^7^	1.89 × 10^8^	1.96 × 10^−10^	5.30 × 10^−10^
GO/EP	0	8.29 × 10^9^	5.15 × 10^11^	1.45 × 10^−10^	8.52 × 10^−11^
	1	9.06 × 10^8^	2.91 × 10^10^	1.60 × 10^−10^	5.82 × 10^−10^
	3	7.02 × 10^8^	8.94 × 10^9^	1.75 × 10^−10^	9.91 × 10^−10^
	7	3.76 × 10^8^	8.44 × 10^9^	1.99 × 10^−10^	4.43 × 10^−9^
	14	1.97 × 10^8^	1.68 × 10^9^	3.07 × 10^−10^	8.32 × 10^−9^
	25	1.42 × 10^8^	1.39 × 10^9^	1.77 × 10^−10^	3.87 × 10^−9^

**Table 2:** EIS fitting parameters of EP and GO/EP coatings before and after UV aging test.

Coating	Time (h)	Rc (Ω·cm^2^)	Rct (Ω·cm^2^)	CPEc (S·sn·cm^−2^)	CPEdl (S·sn·cm^−2^)
EP	0	4.64 × 10^9^	4.06 × 10^10^	4.75 × 10^−11^	9.23 × 10^−11^
	100	2.11 × 10^9^	1.15 × 10^10^	6.68 × 10^−11^	1.62 × 10^−10^
GO/EP	0	8.29 × 10^9^	5.15 × 10^11^	1.45 × 10^−10^	8.52 × 10^−11^
	100	2.21 × 10^9^	1.26 × 10^10^	1.14 × 10^−10^	2.71 × 10^−10^

## Data Availability

The original contributions presented in the study are included in the article and Supplementary Materials; further inquiries can be directed to the corresponding author.

## Supplementary Materials

The following supporting information can be downloaded at https://www.mdpi.com/article/10.3390/nano16191275/s1. Figure S1: Nyquist plots of the EP and GO/EP coatings after different immersion times; Figure S2: Nyquist plots of the EP and GO/EP coatings before and after UV aging; Figure S3: the surface roughness of EP and GO/EP coating before and after UV aging.

## References

[1] Zhang T., Zhang T., He Y., Wang Y., Bi Y. (2023). Corrosion and aging of organic aviation coatings: A review. Chin. J. Aeronaut..

[2] Zeng H., Yang Y., Zeng M., Li M. (2021). Effect of dissolved oxygen on electrochemical corrosion behavior of 2205 duplex stainless steel in hot concentrated seawater. J. Mater. Sci. Technol..

[3] Sun Y., Fu D., Zhu Y., Bai Z., Yuan S., Chang Y., Meng L., Wang H. (2022). A novel anti-oxygen composite coating and its corrosion resistance mechanism. Corros. Sci..

[4] Zhou X., Huang H., Zhu R., Chen R., Sheng X., Xie D., Mei Y. (2020). Green modification of graphene oxide with phytic acid and its application in anticorrosive water-borne epoxy coatings. Prog. Org. Coat..

[5] Wang N., Gao H., Zhang J., Qin Y., Wang D. (2019). Phytic acid intercalated graphene oxide for anticorrosive reinforcement of waterborne epoxy resin coating. Polymers.

[6] Pirhady Tavandashti N., Ghorbani M., Shojaei A., Mol J.M.C., Terryn H., Baert K., Gonzalez Garcia Y. (2016). Inhibitor-loaded conducting polymer capsules for active corrosion protection of coating defects. Corros. Sci..

[7] Cho S., Chiu T.M., Castaneda H. (2019). Electrical and electrochemical behavior of a zinc-rich epoxy coating system with carbon nanotubes as a diode-like material. Electrochim. Acta.

[8] Shen W., Feng L., Liu X., Luo H., Liu Z., Tong P., Zhang W. (2016). Multiwall carbon nanotubes-reinforced epoxy hybrid coatings with high electrical conductivity and corrosion resistance prepared via electrostatic spraying. Prog. Org. Coat..

[9] Al-Saadi S., Raman R.K.S., Anisur M.R., Ahmed S., Crosswell J., Alnuwaiser M., Panter C. (2021). Graphene coating on a nickel-copper alloy (Monel 400) for microbial corrosion resistance: Electrochemical and surface characterizations. Corros. Sci..

[10] Chen S., Cai W., Piner R.D., Suk J.W., Wu Y., Ren Y., Kang J., Ruoff R.S. (2011). Synthesis and characterization of large-area graphene and graphite films on commercial Cu–Ni alloy foils. Nano Lett..

[11] Sanjid A., Anisur M.R., Singh Raman R.K. (2019). Durable degradation resistance of graphene coated nickel and Monel-400 as bi-polar plates for proton exchange membrane fuel cell. Carbon.

[12] Wu Y., Wu Y., Sun Y., Zhao W., Wang L. (2024). 2D Nanomaterials Reinforced Organic Coatings for Marine Corrosion Protection, State of the Art, Challenges, and Future Prospectives. Adv. Mater..

[13] Haddadi S.A., Ramazani S.A.A., Mahdavian M., Arjmand M. (2021). Epoxy nanocomposite coatings with enhanced dual active/barrier behavior containing graphene-based carbon hollow spheres as corrosion inhibitor nanoreservoirs. Corros. Sci..

[14] Zhang F., Yang K., Liu G., Chen Y., Wang M., Li S., Li R. (2022). Recent advances on graphene, Synthesis, properties and applications, Composites Part A. Appl. Sci. Manuf..

[15] Song S., Fan X., Yan H., Cai M., Huang Y., He C., Zhu M. (2023). Facile fabrication of continuous graphene nanolayer in epoxy coating towards efficient corrosion/wear protection. Compos. Commun..

[16] Cui G., Bi Z., Zhang R., Liu J., Yu X., Li Z. (2019). A comprehensive review on graphene-based anti-corrosive coatings. Chem. Eng. J..

[17] Huang S., Kong G., Yang B., Zhang S., Che C. (2020). Effects of graphene on the corrosion evolution of zinc particles in waterborne epoxy zinc-containing coatings. Prog. Org. Coat..

[18] Mao Y., Liu S., Liu S., Wu G., Liu Q., Du X. (2024). Synergistic Effect of CNT and N-Doped Graphene Foam on Improving the Corrosion Resistance of Zn Reinforced Epoxy Composite Coatings. Polymers.

[19] Zhu X., Yan Q., Cheng L., Wu H., Zhao H., Wang L. (2020). Self-alignment of cationic graphene oxide nanosheets for anticorrosive reinforcement of epoxy coatings. Chem. Eng. J..

[20] Ramezanzadeh B., Moghadam M.H.M., Shohani N., Mandavian M. (2017). Effects of highly crystalline and conductive polyaniline/graphene oxide composites on the corrosion protection performance of a zinc-rich epoxy coating. Chem. Eng. J..

[21] Lei Y., Liu Y., Liu Q., Xu Z., You B., Mo X., Wu L., Man H., Ma W., Wang H. (2023). Nacre-mimetic Strategy to Fabricate Waterborne FrGO/ZREoxy Coatings with Dual Corrosion Protection. ACS Appl. Mater. Interfaces.

[22] Zhan Y., Chen Y., Dong H., Li Y., Sun A., Chen X., Yang X., Zhu F., Jia H. (2024). In situ growth of flower-like ZnO onto graphene oxide for the synergistically enhanced anti-corrosion ability of epoxy coating. Ceram. Int..

[23] Guan J., Du X. (2024). Improving the anti-corrosive performance of epoxy/zinc composite coatings with N-doped 3D reduced graphene oxide. Prog. Org. Coat..

[24] Li X., Shi H., Hong R., Peng Y., Liu Z., Xiao B., Yang S., Xu C., Yang Z., Hu Y. (2024). Phytic acid-doped polypyrrole/graphene oxide nanofillers for the preparation of self-healing anticorrosion coatings with the synergistic physical barrier, passivation and chelating effect. Colloids Surf. A Physicochem. Eng. Asp..

[25] Guan J., Wang X., Zou J., Yang J., Du X. (2023). Significant promotion of the anti-corrosive performance of epoxy zinc-containing coating via in situ reduction of graphene oxide and simultaneous graft it with polyamide curing agent. Diam. Relat. Mater..

[26] Ding X., Wang Q., Xu X., Nie X., Xu X., Li J. (2024). A novel epoxy resin composite coating containing polyaniline modified GO with triple anti-corrosion effects of barrier, passivation and corrosion inhibition. Prog. Org. Coat..

[27] Wan H., Min W., Geng L., Li Y., Song D.-D., Chen C. (2025). Design a new type of composite coating of functionalized graphene oxide-composite pyrrhotite/ epoxy resin with excellent hydrogen barrier performance. Corros. Sci..

[28] Yang N., Das C.S., Xue X., Li W., Dai J.-G. (2022). Geopolymer coating modified with reduced graphene oxide for improving steel corrosion resistance. Constr. Build. Mater..

[29] Li H., Zhang Q.-H., Meng X.-Z., Yan H.-J., Hu H.-S., Wu L.-K., Cao F.-H. (2024). Active/ passive protection and anti-UV waterborne epoxy coatings based on low defect functionalized PDA-GO-CeO_2_ material for excellent corrosion control. Chem. Eng. J..

[30] Lai X., Hu J.F., Qu J.Q. (2024). Rational design of photothermally activated self-healing coating with UV resistance towards smart corrosion protection. Prog. Org. Coat..

[31] Sun Y., Yuan S.C., Fan W.H., Lin D., Zhang K., Bai Z.H., Zhu Y.J., Wang H.Y. (2023). A smart composite coating with photothermal response, anti-UV and anti-corrosion properties. Chem. Eng. J..

[32] Sarkar S., Singh A.K., Kulkarni A.R. (2024). In situ O_2_-releasing quantum dot composites with enhanced radical scavenging behaviour, biosafety, and broadband UV shielding applications. Mater. Today Chem..

[33] Hong Q., Xia Y., Wang C., Li Y., Shen Q., Yang H. (2026). Heterogeneous assembly of zirconium-doped CeO_2_ and boron nitride nanosheets as functional nanofillers for excellent corrosion protection and enhanced UV resistance of epoxy nanocomposite coatings. Appl. Surf. Sci..

[34] He S., Wei G., Zhang Z., Yang L., Lin Y., Du L., Du X. (2023). Incorporation of Graphene Oxide Modified with Polyamide Curing Agent into the Epoxy–Zinc Composite Coating for Promoting Its Corrosion Resistance. Polymers.

[35] (2006). Paints and Varnishes—Pull-Off Test for Adhesion.

[36] (2022). Standard Test Method for Linearly Reciprocating Ball-on-Flat Sliding Wear.

[37] Xiao M., Zhang Y., Xiao X., Dai L., Wu Q.-Y., Liu S., Gu L. (2025). Visualizing the macroscale dispersion of graphene based sheets in epoxy anticorrosive coatings by fluorescence quenching. Chem. Eng. J..

[38] Pourhashem S., Vaezi M.R., Rashidi A., Bagherzadeh M.R. (2017). Distinctive roles of silane coupling agents on the corrosion inhibition performance of graphene oxide in epoxy coatings. Prog. Org. Coat..

[39] Narozny M., Zakowski K., Darowicki K. (2018). Application of electrochemical impedance spectroscopy to evaluate cathodically protected coated steel in seawater. Constr. Build. Mater..

[40] Yimyai T., Crespy D., Rohwerder M. (2023). Corrosion-Responsive Self-Healing Coatings. Adv. Mater..

[41] Zhou Z., Wang Z., Fan X. (2025). Enhanced corrosion and UV resistance of double-layer structural epoxy coatings reinforced with graphene oxide/carbon nanotubes hybrids. Constr. Build. Mater..

[42] Shi C., Yu Q., Luo J., Wang J., Du J., Qian J., Len C., Pan A. (2026). Controllable construction and durability application research of graphene based high durability surface protective coatings. Prog. Org. Coat..

[43] (2018). Paints and Varnishes—Determination of Resistance to Liquids—Part 2: Water Immersion Method.

[44] Pei W.L., Pei X.L., Xie Z.Z., Wang J.M. (2024). Research progress of marine anti-corrosion and wear-resistant coating. Tribol. Int..

[45] Zhang Y., Si C., Zhang Z., Li L., Fan X., Zhu M. (2024). Hierarchical design of microcapsules-based epoxy resin coating enhanced with Ti_3_C_2_T_x_ for improving thermal, tribological, anti-corrosive performance. Carbon.

