# Supplementary material for: Facile Enhancement of Corrosion, Wear and UV Aging Resistance of Epoxy Composite Coatings via Graphene Oxide Incorporation
Source: Nanomaterials (Basel). 2026 Oct 8;16(19):1275. doi: 10.3390/nano16191275 (PMC13666659; doi:10.3390/nano16191275)
Supplement: Supplementary file 1 [file nanomaterials-16-01275-s001.zip › nanomaterials-4579008-supplementary.pdf]

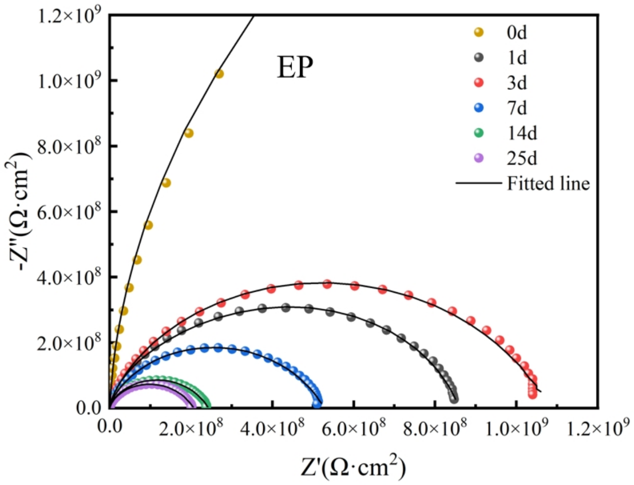

Figure S1. Nyquist plots of the EP and GO/EP coatings after different immersion times.

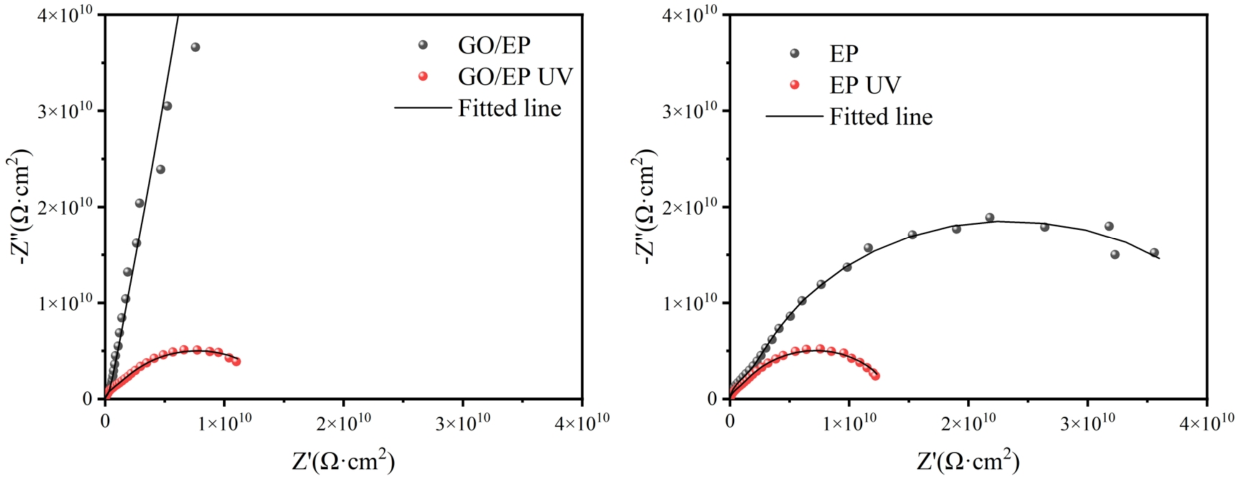

Figure S2. Nyquist plots of the EP and GO/EP coatings before and after UV aging.

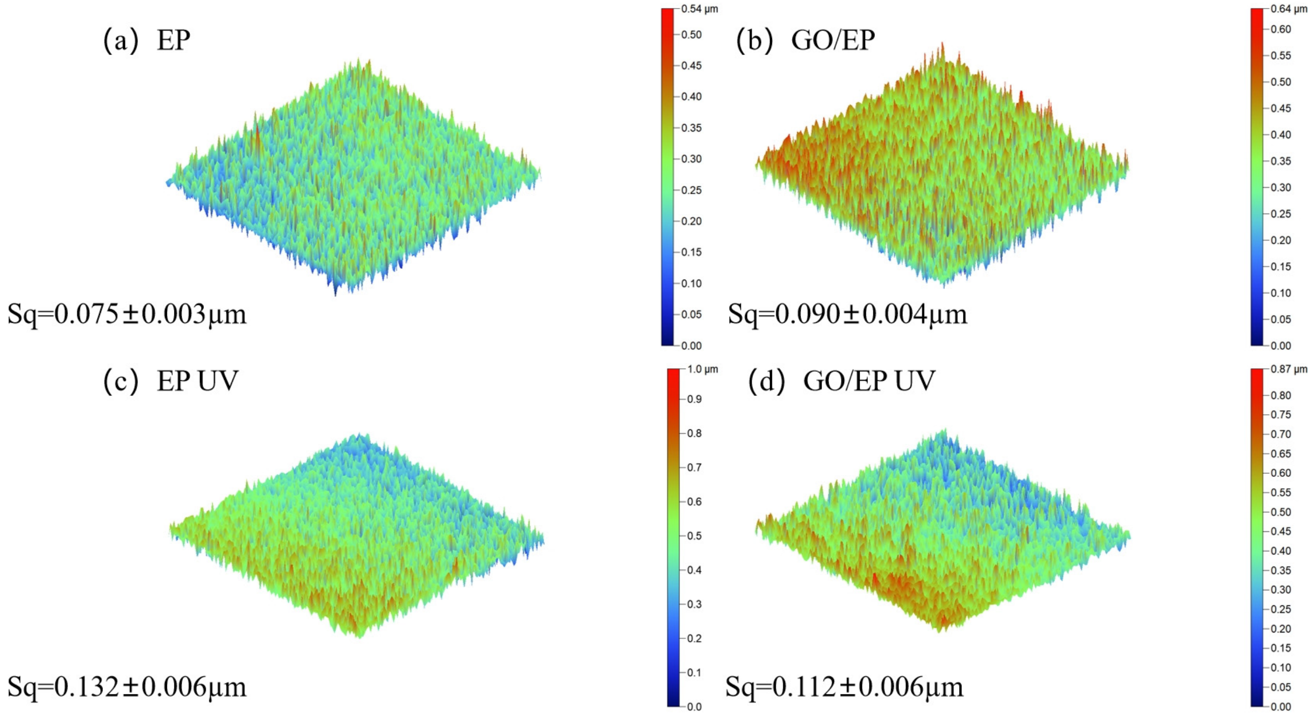

Figure S3. The surface roughness of EP and GO/EP coating before and after UV aging.
